# Exploring Flood Response Challenges, Training Needs, and the Impact of Online Flood Training for Lifeguards and Water Safety Professionals in South Africa

**DOI:** 10.3390/ijerph20166573

**Published:** 2023-08-13

**Authors:** Amy E. Peden, Adrian Mayhew, Shayne D. Baker, Mziwoxolo Mayedwa, Colleen J. Saunders

**Affiliations:** 1 School of Population Health, University of New South Wales (UNSW), Kensington, NSW 2052, Australia; 2 College of Public Health, Medical and Veterinary Sciences, James Cook University, Townsville, QLD 4811, Australia; 3 Surf Life Saving Great Britain, Buckland House, Park 5, Harrier Way, Sowton, Exeter EX2 7HU, UK; amayhew@slsgb.org.uk; 4 International Life Saving Federation—Rescue Commission, 3010 Leuven, Belgium; shayne.d.baker@gmail.com; 5 School of Education, University of Southern Queensland, Toowoomba, QLD 4350, Australia; 6 Department of Information Systems, University of the Western Cape, Bellville 7535, South Africa; mmayedwa@gmail.com; 7 Drowning Prevention Committee, Lifesaving South Africa, Durban 4001, South Africa; colljsaunders@gmail.com; 8 Division of Emergency Medicine, University of Cape Town, Cape Town 7700, South Africa

**Keywords:** drowning, disaster, risk reduction, emergency response, safety, injury, education, emergency, first responder, interoperability

## Abstract

Flooding is a significant cause of human and economic loss in the African region, including in South Africa. Flood mitigation and response in South Africa is challenging due to a range of environmental, infrastructure, and policy constraints. Lifeguards represent a potential additional workforce to bolster flood mitigation and response. This study aimed to explore the feasibility and acceptability of online flood safety training for water safety professionals in South Africa, as well as assess the current flood response capacity and future needs of this group. Online surveys were completed by a convenience sample of South African water safety professionals (including lifeguards) pre-and post a series of four online flood training workshops. Free text responses were thematically coded and flood knowledge was compared between the pre-and post-workshop survey respondents. Sixty-eight responses were analysed (64.7% pre-workshop phase; 63.2% male, 29.4% aged 50–59 years). A range of challenges in flood mitigation and response were identified including equipment, training, and a lack of government support. However, positives were also identified including respondents’ willingness to assist in flood emergencies and good cooperation with neighbouring countries and across the region. Opportunities for better cross-municipal and government communication were discussed. In times of crisis, or in resource poor settings, water safety professionals can bolster traditional flood mitigation and response capacity. Opportunities exist to harness this willingness, but also improve cross-governmental and municipal knowledge sharing to improve future flood mitigation and response efforts in South Africa.

## 1. Introduction

The African continent is significantly impacted by floods, with more than 27,000 fatalities due to flooding in the region between 1950 and 2019 [1,2]. In Africa, rural settlements in riverine and coastal areas are at risk of flooding, while inadequate drainage increases flood risk for residents of urban areas [3]. Across Africa, the number of climate change-induced extreme weather events has increased significantly in the most recent decade (2010–2020) [4], and the number and severity of flood events, in particular, is increasing [1,5]. Such trends indicate a need to improve monitoring, modelling, and communication of the flood hazard in the African region.

Flooding is also a significant concern in South Africa, with the annual risk of flooding estimated to be 83% [6]. Between 1980 and 2010, 77 flood disaster events are estimated to have claimed the lives of 1068 people [6]. Flood risk in South Africa spans riverine flooding, coastal flooding, and inundation of urban areas due to inadequate drainage [4,7]. More recently, flooding in the province of KwaZulu-Natal in mid-April 2022 claimed the lives of 443 people and displaced an estimated 40,000 people after their homes were swept away by floodwaters [8]. 

Flood mitigation and response in South Africa is challenging for a range of reasons. From an environmental perspective, the development of poor-quality housing on areas prone to flooding, blocked drainage channels in urban areas, poorer access to services, and inadequate sewerage and stormwater infrastructure all exacerbate flood-risk [9]. From a policy perspective, resource challenges and limitations in disaster management structures at the district level lead to civil defence authorities, such as fire and rescue and police, becoming overburdened and reducing efficiency in flood response [6]. 

Lifeguards, who typically respond to water emergencies, represent a potential asset for flood-prone areas without adequate resources. Research from the United Kingdom (UK) and Australia has found lifeguards to be willing and able to assist in flood mitigation and response, particularly as an asset to assist other emergency services [10], albeit with the appropriate prior training and equipment. This study is the only research to date which has conducted research with this cohort as it relates to flood mitigation and emergency response due to disasters. Therefore, there is a need to bolster understanding of the challenges and opportunities of leveraging this workforce during flood disaster. 

Similarly, little research appears to have been published on the topic of flood training assessment, when compared to literature regarding flood susceptibility [11,12] and flood hazard risk assessment [13,14]. One study proposes an expert system as a means to evaluate and improve civil defence flash flood training capabilities in Saudi Arabia, finding this approach may help improve training to better anticipate locally identified flood risks [15]. Other studies have shown technological approaches, such as virtual reality [16] and system dynamics [17], can be used in emergency response training, including for flood disasters. However, no prior studies have examined flood response training among lifeguards in South Africa. 

The increasing flood threat in South Africa, ongoing resource limitations, and challenges associated with flood mitigation and response, coupled with emerging literature identifying lifeguards as a potential resource in flood mitigation and response [10], suggest that there is an opportunity to upskill lifeguards and water safety professionals (henceforth referred to as water safety professionals) to assist in flood mitigation and response. From the perspective of South Africa-based water safety professionals, this study aimed to document the challenges in flood mitigation and response, to describe the flood training needs of this cohort, and to explore the feasibility and acceptability of online flood safety training.

## 2. Materials and Methods

This quasi-experimental cohort study analysed a convenience sample of responses to online, anonymous surveys completed before and after a series of four online workshops around flood disaster mitigation and response.

### 2.1. Background to Training

Prior to the conduct of the online training described in this study, the need for education on flood emergency awareness and skill development had been identified based on first-hand accounts from practitioners [10], and due to increasing flood risk across the globe [18]. As a result of this identified need, authors SB and AM (as well as Professor Mike Tipton and Dr Patrick Morgan) facilitated a one-day flood disaster workshop prior to the World Conference on Drowning Prevention in Durban, South Africa, in 2019. Immediately prior to this event, the neighbouring country of Mozambique had experienced significant flooding [19] and this provided a timely reminder of the impact of flooding in low- and middle-income countries (LMICs). Delegates at the workshop were drawn from seven countries and expressed an interest to learn more about flood safety and the ways in which trained lifesavers could contribute to preventing drowning in flood disasters.

Following the workshop, an invitation was extended from the President of Lifesaving South Africa (LSA) to conduct a series of flood disaster mitigation and response workshops in South Africa. Prior to implementing these in-person workshops, the COVID-19 pandemic and related restrictions impacted the feasibility of in-person workshops and an alternative offer was made by author SB to facilitate a series of online workshops. LSA agreed to host the events and promote the events throughout the African continent, Royal Life Saving Society Commonwealth, and International Life Saving (ILS) Federation member organisations via email communications and social media posts.

### 2.2. Training

As the online format presented challenges with respect to the knowledge and skills that could be taught, it was agreed that the initial ninety-minute session would aim to share Emergency Management Principles used in previous flood disaster management training [10], and to undertake a pre-workshop survey of attendees to gauge the interests, existing knowledge and expertise of workshop participants.

The outcomes Identified from the pre-workshop survey were used to develop a further three online workshops of ninety-minute duration which covered the modules shown in Table 1:

The following three workshops each included a 30-min presentation from experienced local individuals to incorporate an understanding of the local context. Specifically, these included the Deputy Director of a South African provincial disaster management: Mitigation unit, a field and operations manager from Mozambique, emergency response personnel, and a layperson impacted by flooding. These were supplemented by more formal teaching modules.

### 2.3. Participant Recruitment

**Figure 1:**
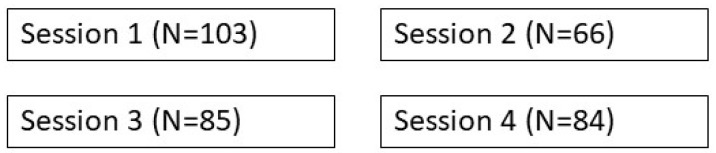
Study population by number of online workshop sessions.

The study population comprised a convenience sample of all the participants (excluding presenters) who attended any of the four sessions. Study population numbers by session are shown in Figure 1.

Only 15 (7%) of participants attended all four sessions. Workshop participants were recruited by wide promotion through the LSA website, email mailing lists and social media platforms. Interested participants were asked to pre-register for the workshop via an email address actively monitored by LSA head office. Registrants were sent an email including information about this study, an invitation to participate in the study, and a link to the pre-workshop survey to be completed ahead of the first webinar. Similarly, all registered workshop participants were invited to participate in the post-workshop survey at the completion of the final webinar. The surveys collected voluntary, informed consent from participants prior to commencing the survey. Participation in the workshops was not restricted by geographical location of the registered participant; however, only South African participants were included in the study sample.

### 2.4. Survey Design and Development

Surveys were developed by authors AP, SB, and AM in word and then transferred into Qualtrics (Provo, UT, USA) survey design software. The pre-workshop survey included 47 questions across seven sections: (i) demographics and personal flood experience, (ii) organisational flood mitigation and response capacity, (iii) country-level flood mitigation and response capacity, (iv) flood response/management resources, (v) Sendai Framework Awareness and Flood Management Knowledge, (vi) Funding, and (vii) Flood Knowledge (Supplementary file S1). 

The post-workshop survey included 37 questions across six sections: (i) demographics and personal flood experience, (ii) individual participants’ response to training, (iii) individual respondent’s knowledge, (iv) organisational flood mitigation and response capacity, (v) country-level Flood Mitigation and Response Capacity, and (vi) any additional feedback (Supplementary File S2).

Surveys were pilot-tested for comprehension, clarity, and face validity before they went live. Due to the piloting minor changes were made to the order of questions and the question design.

### 2.5. Data Cleaning, Coding, and Analysis

Raw data were downloaded from Qualtrics in SPSS format and analysed in IBM SPSS V25 (Chicago, IL, USA). The pre-workshop survey received a total of 68 responses, a response rate of 66%. After removal of 18 responses from respondents not residing in South Africa and a further 6 largely incomplete responses, a total of 44 responses were retained for analysis. For the post-workshop survey, 43 responses were received, a response rate of 51%. After removal of 14 non-South African resident respondents and 5 incomplete responses, 24 post-workshop surveys were analysed. 

Survey responses for the pre-workshop and post-workshop surveys were analysed separately when examining respondent characteristics and the impact of training on flood knowledge. As respondents’ characteristics were similar in both the pre- and post-workshop respondent groups, responses to questions regarding challenges in flood mitigation and response were combined for ease of presentation. 

Age of respondents were coded into the following groups: 20–29 years; 30–39 years; 40–49 years; 50–59 years; and 60–69 years. Five-point Likert scales (excellent, very good, neutral, poor, and very poor) for the assessment of workshop topics were condensed into three combining ‘excellent’ and ‘very good’ into one category and ‘poor’ and ‘very poor’ into another. Free text and open-ended responses were thematically coded for ease of analysis by two researchers using an inductive, multi-phase approach described by Braun and Clarke [20]. Select quotes were included verbatim to contextualise responses; these were presented alongside the respondent’s sex, age group, and the type of organisation they were representing.

### 2.6. Human Research Ethics

This study received human research ethics approval from the University of Southern Queensland (H21REA073 V1).

## 3. Results

In total, 68 valid responses were analysed, with 44 from the pre-workshop phase (64.7% of all surveys).

### 3.1. Participant Demographics

Males accounted for a similar proportion of both the pre- (63.6%) and post-workshop (62.5%) survey respondents. The mean age of the pre- (48.0 years [SD = 11.5]) and post-workshop (50.5 years [SD = 11.3]) survey respondents was also similar. Respondents most commonly represented volunteer organisations in both pre- (n = 13; 29.5%) and post-workshop (n = 10; 41.7%) survey respondent groups, most commonly in strategic or middle management positions. A slightly higher proportion of respondents had previously responded to flooding in South Africa in the post-workshop survey (54.5%), when compared to the pre-workshop survey (47.7%) (Table 2).

When asked in the pre-workshop survey what prior flood response/flood management training respondents had undertaken, the most commonly reported training was in lifesaving/lifeguarding (n = 13; 29.5%). Organisational roles in flood mitigation and response as represented among pre-workshop survey respondents most commonly comprised search and rescue (n = 27; 61.4%) and community preparedness (n = 15; 34.1%). Respondents also reported other tasks such as closure of beaches and rivers, education, and disaster risk reduction.

### 3.2. Flood Mitigation and Response Capacity and Challenges

#### 3.2.1. At the Individual Level

A range of individual challenges were identified by respondents to the pre-workshop survey. The most common challenges identified were related to equipment (n = 15; 34.1%), training (n = 13; 29.5%), and human capacity (n = 9; 20.5%). After they had participated in the training, respondents were asked, via the post workshop survey, what challenges remained. Just over half of respondents indicated practical training was now a key challenge (n = 13; 54.2%), followed by equipment and funding (n = 10; 41.7% respectively) concerns. 

Interoperability (n = 6; 25.0%) and a lack of government support (n = 4; 16.7%) were further identified as challenges in the post-workshop survey, summed up by one respondent:

*“[There is a] lack of communication and coordination by local government structures with communities prone to flood disasters and flooding and rescue [are] not prioritised financially”*.

(Female, 50–59 years of age, employee of non-Government organisation)

When asked what other organisations respondents collaborate with on flood management/response, these included emergency response (n = 13; 19.1%), police (n = 6; 8.8%), fire (n = 4; 5.9%), and weather/meteorological services (n = 2; 2.9%). Other organisations mentioned included Lifesaving South Africa, Mountain Rescue, White Water Rescue, Insurance companies, National Sea Rescue Institute, and Swimming South Africa. It was noted that weather meteorology services assist with pre-flood planning in South Africa via media warnings, weather forecasts, and information sharing. They also assist in flood response via live satellite imagery and forecasting, as well as continuous monitoring of weather and providing feedback to rescuers.

#### 3.2.2. At the Organisational Level

Among pre-survey respondents, organisational flood response and management capacity were predominantly described as good (n = 15; 34.1%) or fair (n = 13; 29.5%). Just four respondents (9.1%) described capacity as excellent. Respondents to the pre-survey indicated that the most common organisations, in addition to their own, who were involved in flood management and response were fire and rescue (n = 32; 72.7%), emergency services (n = 30; 68.2%), volunteers (n = 26; 59.1%), police (n = 25; 56.8%), and local government (n = 23; 52.3%). 

With respect to their own organisation, the foremost organisational strength identified was an organisation’s people (n = 14; 31.8%), with one respondent highlighting compassion:

*“Our teams are compassionate people who take pride in helping others in need”*.

(Female, 50–59 years, volunteer)

Another indicated the willingness of people to ensure safety:

*“The willingness of the available staff to ensure the safety of the community as well as the large community outpouring of volunteers during such incidents is positive”*.

(Female, 20–29 years, Government)

Human resources were also valued due to willingness to improve their skills and knowledge as one respondent said:

*“Lots of individuals who are eager to learn and respond”*

(Male, 20–29 years, volunteer)

Training was identified as the second leading organisational strength (n = 10; 22.7%) with:

*“Teaching and training opportunities on all levels of response and management levels.”*.

(Male, unknown age, other—management)

However, one respondent cautioned about the transferability of training from traditional lifeguard environment to flood response:

*“As lifeguards, we are well training as far as how to save a life. however, when it comes to how to save a life during flooding is a different situation. For instance, performing a rescue in the surf, the pool is easy as there is no debris that you must be aware of during the rescue”*.

(Male, 40–49 years, non-Governmental organisation)

In the pre-workshop survey, organisational challenges commonly identified among respondents were access to adequate resources (n = 12; 27.3%), unqualified people (n = 8; 18.2%), and safety in the environment (n = 3; 6.8%). The issue of unqualified people was summed up by two respondents as:

*“Lifesaving training is limited as it does not include flood management”*

(Male, 40–49 years, non-Governmental organisation)

and

*“Relying on outdated techniques and information- unable to integrate with other organisations fulfilling a similar role with newer methods/knowledge”*.

(Male, 20–29 years, volunteer)

With respect to safety concerns two respondents noted unique challenges such as:

*“ability to swim”*

(Male, 50–59 years, Other- instructor/assessor)

and

*“illegal electricity connected on the flooded areas”*.

(Female, 30–39 years, Government)

#### 3.2.3. At the Country Level

On a national scale, respondents indicated several strengths in flood mitigation and response including people (n = 8; 18.2%), resources (n = 8; 18.2%), and interoperability (n = 3; 6.8%). With respect to interoperability one respondent noted there are:

*“Coordinated efforts from National Disaster Management Centre, Provincial and Local”*.

(Female, 30–39 years, Government)

Another indicated:

*“Well coordinated disaster management plans involving all spheres of government and non-governmental organizations involved in lifesaving and water safety”*.

(Female, 50–59 years, non-Governmental organisation)

This even extends internationally as one respondent said:

*“Local and international arrangements for multi-agency cooperation”*.

(Male, 30–39 years, Government)

These strengths are not without challenges, as one respondent said:

*“we have an excellent workforce, that needs training to function optimally”*.

(Female, 50–59 years, Volunteer)

As another noted, there is government willingness to assist, but timeliness is a problem:

*“The fact that the national government does try to assist the victims of such incidents [is a strength]. The flood response is quite delayed or outdated though.”*.

(Female, 20–29 years, Government)

Expanding on these challenges, respondents were asked to record challenges South Africa faced at a national level with respect to flood mitigation and response. Commonly reported challenges included training (n = 10; 14.7%), infrastructure and planning (n = 9; 13.2%), equipment (n = 8; 11.8%), interoperability (n = 6; 8.8%), and responsiveness (n = 5; 7.4%). As several respondents noted, these challenges are often interlinked, for this respondent interoperability impacts responsiveness:

*“Inability to co-ordinate a unified, quick and effective response”*

(Male, 60–69 years, not-for-profit)

Another indicated responsiveness is impacted by interoperability and poor infrastructure and planning:

*“Sometimes the responses are not well coordinated due to mushrooming informal settlements in low lying flood line areas. Predominantly rural villages that are far from service points”*.

(Female, 50–59 years, non-Government organisation)

Interoperability appears to be a big source of tension as one respondent indicated:

*“Different state departments that do not want to work together and is unable to understand that there are overlaps with respect to responsibilities, mandates and service delivery—every department should be involved. Working in silos to hide behind “it is not our responsibility” will not solve the flooding problems and the related threats. Additionally, not including and listening to the most skilled and knowledgeable people in the planning, development and execution of flood response and management plans and structures”*.

(Male, unknown age, other—management)

Several respondents expressed concern regarding a lack of modern approaches to the issue of flood mitigation and response: As one respondent said:

*“[There is an] outdated view of future flooding incidents and the effect of climate change on the country’s environment”*

(Female, 20–29 years, Government)

and another commented that there is:

*“Insufficient disaster risk resilience (DRR) approach to flood risk from a developmental point of view”*.

(Male, 30–39 years, Government)

When asked how floods are managed at the local, regional, and national level, it was indicated that:

*“There is a national framework and/or strategy that gets implemented at provincial and local level to ensure consistency and coherence”*.

(Female, 50–59 years, non-Government organisation)

However, many other respondents were critical about the management of flood response including response times, coordination, and capacity:

*“Very bureaucratically which leads to long delays in response”*.

(Male, 50–59 years, Strategic manager)

*“Very slowly and very poorly with very poor communication and co-ordination”*

(Male, 60–69 years, Strategic manager)

*“Not really sure, as all this seems very vague at this stage. Local and regional authorities mainly rely on Police, Military and Fire Fighting Departments to handle that. Very few private or NGO’s are involved”* .

(Male, unknown age, other—management)

Of the 20 responses to the question regarding whether neighbouring countries assist in flood management/response, 10.3% (n = 7) indicated yes. This assistance ranged from neighbouring countries providing human resources to sponsorship of drowning prevention and water safety awareness campaigns. It was also acknowledged by several respondents that South Africa often assists other countries, such as Mozambique, via the Southern African Development Community (SADC) integrated disaster management programme.

### 3.3. Equipment, Standards, Funding, and Training 

When asked about the flood management equipment they have access to, responses ranged from human resources only, to floatation devices, small first aid kits, water pumps and throw bags, and to powered craft such as inflatable rescue boats. One respondent indicated they had access to:

*“Inflatables, Fire and rescue equipment, army and air force standby”*.

(Male, 60–69 years, volunteer)

However, many indicated there were equipment shortages such as one respondent who stated they had access to *“Surf Lifesaving Equipment”* (Male, 50–59 years, Government) while another indicated:

*“We have limited number of swift and standing water rescue equipment including 2 rubber ducks and inflatables. This is limited to only 1 of the 5 districts within our jurisdiction”*.

(Male, 30–39 years, Emergency Response)

Six respondents indicated they had no access to equipment for flood response and management. When asked what equipment was needed, personal protective equipment (PPE) and rescue boats were the most commonly mentioned. However, equipment needs were wide reaching as one respondent said:

*“[We need] Everything, from office equipment to response transport (land and water transport) and rescue equipment”*

(Male, unknown age, other—management)

Another highlighted the need for resources to allow for community outreach:

*“Internet connectivity, computers, audio visual presentation equipment, instructors to prepare children and communities through outreach training and education, how to react in such an emergency. Preparation and readiness saves lives”*.

(Male, 60–69 years, not for profit organisation)

Respondents were also asked about standards for flood mitigation and response in their local area. Eight respondents (11.8%) indicated awareness of standards for flood mitigation and response. These responses were most commonly around basic training, incident command and communications. Five respondents (7.4%) indicated there was funding available to assist in purchasing flood rescue and management equipment. 

Respondents indicated that training in swift water rescue (n = 7; 10.3%), lifesaving (n = 5; 7.4%), and disaster management (n = 2; 2.9%) was available. Training needs spanned swimming and public awareness training to mass casualty management and disaster management roles and responsibilities, including simulation exercises. As one respondent said, all forms of training are required:

*“Flood reporting and early warning; flood response coordination, management, search and rescue, “After care” including, what to do in order to prevent flooding it in the future”*

(Male, unknown age, other—management)

As one respondent summed up, what is required is:

*“correct allocation of funding and organised training programmes”*.

(Male, 50–59 years, other—instructor/assessor)

### 3.4. Evaluation of Training

In the post workshop survey, the majority of respondents indicated they had attended all four of the online workshops (n = 15; 62.4%). The vast majority of respondents (n = 21; 87.5%) rated the training they had received as excellent. Training standards modules (69.6% of respondents rating it as excellent) and the flood disaster and future module (65.2% rating it was excellent) were the most highly rated. All respondents to the post workshop survey (n = 24; 100.0%) indicated they would recommend the training to others involved in floods. 

In the post workshop survey, 87.5% of respondents (n = 21) assessed collaboration with other organisations or sectors on flood mitigation and response to be very important, and two thirds of respondents (66.7%) indicated that their participation in the training had provided them with the motivation and confidence to become involved in flood interoperability.

### 3.5. Post-Training Responses and Remaining Training Needs

Post workshop survey respondents were asked if the training had prompted them to make any changes in the way they currently manage and/or respond to flooding, to which 91.7% (n = 22) indicated that it had. Several respondents identified training opportunities:

*“We need to include some of the key introductory points into our entry level lifeguard training”*

(Male, 50–59 years, volunteer)

and

*“Upgrade equipment and formulize better training for rescue personnel”*.

(Male, 50–59 years, Government employee)

Several other respondents identified the need for unity and standardisation in approaches including the need to *“Unify various response organizations”* (Male, 60–69 years, volunteer) and “*standardise the disaster response between the branches”* (Male, 60–69 years, Emergency Response). 

Investment was also identified as an action out of the training, with one respondent noting they would *“Try and convince the higher up authorities to invest more in flood and water rescue”* (Male, 50–59 years, Government employee). 

When asked about remaining training needs, respondents identified a need for practical training as one respondent said:

*“All of them—in a practical sense, however, I would believe my strength would lie in once I have the basics ... further training in strategic disaster management” (Female, 50–59 years, Volunteer). Additionally, context specific training was required such as “Community preparedness for rural areas prone to flooding but far from Emergency Disaster Management interventions services”*.

(Female, 50–59 years, non-Government organisation employee)

## 4. Discussion

Southern Africa is a region susceptible to extreme weather events, including floods and storm surges, which are predicted to increase in frequency and magnitude due to climate change [21]. Despite South Africa being identified as the country in Southern Africa with the highest number of recorded climatological events, it accounts for a small percentage of deaths. This is likely due to lower social vulnerability and more mature adaptation and disaster management strategies compared to other Southern Africa Development Community counterparts [21]. However, challenges in flood mitigation and response remain and, in settings where existing flood mitigation and response may be limited, water safety professionals represent a potential workforce to bolster existing resources [10]. 

In this study, we explored the challenges in flood mitigation and response and the flood training needs among water safety professionals in South Africa. Our study evaluated an online approach to flood safety training, borne out of necessity during COVID-19 pandemic-associated travel restrictions. We found that the provision of flood safety training in an online format is feasible and acceptable to participants, as has been shown in other countries and for other audiences [22]. We did find that flexibility in curriculum design was important and enabled modification of course content based on the local needs and level of knowledge identified in the pre-survey of potential participants. However, hands-on training remained an identified need from participants. Our previous work has shown that surf lifesavers are responding in times of flood prior to having received specific flood response training, which may place them at increased risk of harm [10]. This should be considered during future online training, and emphasis placed on the need for supplementary practical training in conjunction with local role players before responding to flood disasters.

Beyond hands-on, practical training, study participants identified a range of challenges and needs to aid in more effective flood mitigation and response in South Africa. These challenges and needs have also been identified by government and the development community in Southern Africa [23] and include funding, equipment, interoperability, and flood plain management due in part to poor land use practices [24]. Related to land use practices are the unique training issues related to poor infrastructure and informal housing; these include health and safety issues during flood response related to sewage and building material debris such as corrugated iron sheeting [25]. Similarly, awareness of the risk of encounters with escaped wildlife and training on the safe management thereof was highlighted in the April 2022 floods in KwaZulu-Natal [26]. Any hands-on training provided in the future must be tailored to the specific needs of the South African context, and should include consideration of the diversity of localised risks in different regions. 

Many respondents highlighted their interest in, and willingness to, respond to floods as volunteers, a desire to be able to respond appropriately and efficiently. Such desire should be harnessed through practical training and coordination to build capacity that can support formal governmental responses in South Africa and the region.

Respondents also identified good cooperation with neighbouring countries and within the region. This is a positive finding, given transboundary or regional programs have been shown to be vital in Southern Africa to reducing damage and loss of life due to extreme events such as floods [27]. However, respondents did note that within the country, there is a need for improved city to city learning and knowledge exchange, including across different levels of government to improve future flood mitigation and response efforts [28]. A key challenge in flood response in South Africa is that it occurs at multiple levels of government. Flood response is initiated by municipal governance structures where the disaster management function may be incorporated into broader organisational structures and there is wide diversity in the of capacity and resources for flood response. Once a disaster exceeds the capacity of the municipal structures to respond, coordination of the response will escalate to provincial and then national Disaster Management centres. This means that initial response approaches may differ by region and presents a challenge for standardisation and training. In addition, resource challenges in the face of multiple issues such as fires, drought, disruptions in electricity supply, and disease outbreaks present a challenge for prioritising resourcing of flood mitigation efforts [29]. In addition to better knowledge exchange across levels of government, respondents also noted a need for better integration with other emergency services and weather bureaus as a future recommendation for improvement [27]. 

Our study has yielded new knowledge describing the limitation of traditional lifesaving training and equipment as it pertains to assisting in flood disasters. At the same time, this study has shown that lifeguards and water safety professionals are willing to assist and augment existing flood disaster response mechanisms and these professionals represent a currently under-utilised human resource. We have shown the value of online training (supplemented by practical training) for development knowledge in flood evacuation, rescue, and recovery capacity. Many opportunities for future research remain, including investigation of the strategies drowning prevention and lifesaving organisations have developed in response to the Sendai Framework for Disaster Risk Reduction 2015–2030 [30]; identifying gaps in the current strategies of lifesaving organisations to respond to a flood disaster; and comparing a country’s drought preparedness to flood preparedness as there are likely lessons that can be transferred from one climate disaster to another in the context of Southern Africa [31].

### Strengths and Limitations

This study is the first to examine the challenges facing water safety professionals in South Africa in flood mitigation and response, as well as explore the feasibility of online flood safety training for this cohort. This study provides helpful information on the strengths and challenges of water safety professionals in responding to floods and provides recommendations for future research and capacity development. However, there are several limitations associated with this study. Due to the survey design constraints and the anonymous nature of the survey, we were not able to track and link individual respondents across both the pre-and post-surveys. The survey also did not utilise mandatory fields and, as such, there were some questions with missing data which limited analysis. The training and surveys were conducted in English language and may have limited participation from non-English speakers, although it should be noted that the workshops were conducted in English, which is widely spoken in South Africa. Respondents were drawn from a convenience sample, and responses should not be considered to be broadly representative. Although the anonymous nature of the survey may have reduced the likelihood of social desirability or demand characteristic bias in responses [32,33], these limitations are associated with data collected via surveys and should, therefore, be considered. Due to the large number of non-responses to knowledge questions, it was not possible to assess the impact of training on knowledge gain. Though they were excluded from analysis, blank responses to knowledge questions likely also indicated low knowledge or poor understanding of what was being asked of respondents.

## 5. Conclusions

With flooding predicted to increase in severity and frequency due to the effects of climate change, lifesaving personnel may represent an additional asset in flood mitigation and response, particularly in resource poor settings. Our research in surveying water safety professionals in South Africa alongside the provision of flood safety training has identified strengths and challenges in flood mitigation and response. This study identified a valuable group of skilled professionals willing to assist in times of flood, albeit with a need for practical training and formal coordination to build capacity.

## Figures and Tables

**Table 1:** Modules covered in online workshops.

Module Name
Flood disaster and future
Emergency management
The four phases of flooding
Principles of incident command—TEMPOE
Flood hydrology
Operations safety
Training standards
Equipment standards

**Table 2:** Demographic characteristics of respondents across both pre- and post-workshop surveys.

	Pre-Workshop Survey		Post Workshop Survey	
	Number	%	Number	%
Total	44	100.0	24	100.0
Sex				
Female	13	29.5	9	37.5
Male	28	63.6	15	62.5
Prefer not to say	3	6.8	0	0.0
Age group				
20–29 years	4	9.1	0	0.0
30–39 years	8	18.2	6	25.0
40–49 years	9	20.5	5	20.8
50–59 years	13	29.5	7	29.2
60–69 years	9	20.5	6	25.0
Unknown	1	2.3	0	0.0
Type of organisation represented				
Emergency response	7	15.9	2	8.3
Government	11	25.0	5	20.8
Non-Government	5	11.4	5	20.8
Volunteer	13	29.5	10	41.7
Other	8	18.2	2	8.3
Position within organisation				
Lifeguard	3	6.8	4	16.7
Operator/Technician	5	11.4	4	16.7
Middle Manager	13	29.5	4	16.7
Strategic Manager	16	36.4	9	37.5
Other	6	13.6	4	16.7
Previously responded to flooding in your country?				
Yes	21	47.7	13	54.2
No	23	52.3	11	45.8

## Data Availability

Due to ethical constraints, underlying data cannot be shared. Those interested in accessing the data upon reasonable request are asked to please email the corresponding author (a.peden@unsw.edu.au).

## Supplementary Materials

The following supporting information can be downloaded at: https://www.mdpi.com/article/10.3390/ijerph20166573/s1, Supplementary File S1: Community Development and Sustainability in Flood Disaster Management: Pre-workshop series survey; Supplementary File S2: Community Development and Sustainability in Flood Disaster Management: Post-workshop Series Survey.
