# Supplementary material for: Exploring Flood Response Challenges, Training Needs, and the Impact of Online Flood Training for Lifeguards and Water Safety Professionals in South Africa
Source: Int J Environ Res Public Health. 2023 Aug 13;20(16):6573. doi: 10.3390/ijerph20166573 (PMC10454401; doi:10.3390/ijerph20166573)
Supplement: Supplementary file 1 [file ijerph-20-06573-s001.zip › ijerph-2471303-supplementary.pdf]

Supplementary File S1: Community Development and Sustainability in Flood Disaster Management: Pre-workshop Series Survey

Introduction
Thank you for agreeing to complete this pre-workshop survey. De-identified responses to this survey will be used to refine the workshop content and evaluate and improve future iterations of this workshop. This study has received ethical approval from the University of Southern Queensland (H21REA073).
In participating in the survey, there are no anticipated risks beyond normal day-to-day living. However, sometimes thinking about the sorts of issues raised in the questionnaire can create some uncomfortable or distressing feelings. If you need to talk to someone about this immediately, please contact the South African Depression and Anxiety Group (SADAG) on (0800) 121314 of a local hotline in your country.
By answering 'yes' below, you are indicating:
You have read and understood the information document regarding this project.
Have had any questions answered to your satisfaction.
Understand that if you have any additional questions, you can contact the research team.
You are over 18 years of age.
You understand that any data collected may be used in future research activities.
You agree to participate in the project.
Please click yes to start the survey. If you don't wish to participate please exit the survey now by closing the page.

○ Yes (1)

Demographics and personal flood experience
Which country do you live in?

____

Click to write the question text

○ Male (1)

○ Female (2)

○ Non-binary / third gender (3)

○ Prefer not to say (4)

What is your age?

____

What is the type of organisation you work with? [choose most appropriate]

- ○ Government (1)
- ○ Non-government organisation (2)
- ○ Emergency response (3)
- ○ Volunteer (4)
- ○ Other [please specify] (5) ____

What is your position within your organisation?

- ○ Operator/Technician (1)
- ○ Middle Manager (2)
- ○ Strategic Manager (3)
- ○ Other [please specify] (4) ____

Have you responded to flooding in your country before?

- ○ Yes (1)
- ○ No (2)

If yes, how many floods have you been involved in?

____

What are 3 key primary challenges you face in flood response/management?

- ○ Challenge 1 (1) ____
- ○ Challenge 2 (2) ____
- ○ Challenge 3 (3) ____

What flood response/flood management training have you had? [Please also include any training related to lifesaving, drowning or emergency response]

____

____

____

____

____

Organisational Flood Mitigation and Response Capacity

What role(s) does your organisation play in flood management/response in your country? [select all that are relevant]

- ▢ Community Preparedness (1)
- ▢ Search & Rescue (2)
- ▢ Flood Management (3)
- ▢ Other [please specify] (4) ____

How do you rate the strengths of the organisation's flood response/management capacity?

- ○ Excellent (1)
- ○ Good (2)
- ○ Fair (3)
- ○ Poor (4)
- ○ Very Poor (5)

What are the main strengths you see in your organisation's flood response/management?

____

____

____

What are three key challenges your organisation faces in flood response/management?

- ○ Key Challenge 1 (4) ____
- ○ Key Challenge 2 (8) ____
- ○ Key Challenge 3 (9) ____

Country-Level Flood Mitigation and Response Capacity

In addition to your own, what other organisations are involved in flood management/response in your country? [choose as many as are relevant]

- ☐ Police (1)
- ☐ Fire and Rescue (2)
- ☐ Emergency Services (3)
- ☐ Volunteer (4)
- ☐ Local Government (5)
- ☐ Regional Government (6)
- ☐ National Government (7)
- ☐ Other [please specify] (8) ____

What are the main strengths you see in your country's flood response/management?

____

____

____

____

____

What are three key challenges your country faces in flood response/management?

- ○ Challenge 1 (1) ____
- ○ Challenge 2 (2) ____
- ○ Challenge 3 (3) ____

Flood Response/Management Resources

What equipment do you have for flood response/flood management?

____

____

____

What equipment do you require?

____

____

____

What training is available for flood response/flood management?

____

____

____

____

What training do you require?

____

____

____

____

What assets are available for flood response/flood management?

____

____

____

What assets do you require?

____

____

____

Sendai Framework Awareness and Flood Management Knowledge

Are you familiar with the Sendai Framework?

○ Yes (1)

○ No (2)

○ Don't know (3)

In your own words, what do you think the Sendai Framework is

____

____

____

How is your work aligned to the Sendai Framework?

____

____

____

Do your neighbouring countries assist in flood management/response?

- Yes (1)
- No (2)
- Don't know (3)

If they do assist, how?

____

____

____

How are floods managed between the local, regional and national level?

____

____

____

Are there any agreements in place regarding working collaboratively (inter-operability), such as capability, communications, protocols, between disaster managers, government departments and responders?

- Yes (1)
- No (2)
- Don't know (3)

If there are agreements in place, what are they?

____

____

____

What other organisations do you work with on flood management/response?

○ Weather/Meteorology (1)

○ Emergency Response (2)

○ Police (3)

○ Fire (4)

○ Other [please specify] (5) ____

Does the weather or meteorology office assist with pre-flood planning?

○ Yes (1)

○ No (2)

○ Don't know (3)

If they do assist with pre-flood planning, how?

____

____

____

Does the weather or meteorology office assist with emergency flood response?

○ Yes (1)

○ No (2)

○ Don't know (3)

If they do assist with emergency flood response, how?

____

____

____

Are there standards for flood mitigation and response (such as operations, responsiveness, communications etc)?

- ◯ Yes (1)
- ◯ No (2)
- ◯ Don't know (3)

If there are standards, what are they?

____

____

____

____

____

Funding

Is there funding to assist in purchasing flood rescue and flood management equipment?

- ◯ Yes (1)
- ◯ No (2)
- ◯ Don't know (3)

How much is spent on flood disaster and flood relief programs in your country?

____

Flood Knowledge

Are you aware of the coastal sea level rises that are predicted in this century?

- ◯ Yes (1)
- ◯ No (2)
- ◯ Don't know (3)

If yes, do you know by what height sea levels are expected to rise?

____

Are you aware of any plans for mitigation or response to coastal inundation?

- ○ Yes (4)
- ○ No (5)

Name the 5 flood rescue modules

- ○ Module 1 (1) ____
- ○ Module 2 (2) ____
- ○ Module 3 (3) ____
- ○ Module 4 (4) ____
- ○ Module 5 (5) ____

Name the 4 phases of floods

- ○ Phase 1 (1) ____
- ○ Phase 2 (2) ____
- ○ Phase 3 (3) ____
- ○ Phase 4 (4) ____

Name 3 emergency flood principles

- ○ Principle 1 (1) ____
- ○ Principle 2 (2) ____
- ○ Principle 3 (3) ____

Name 2 hydrological flood features

- ○ Hydrological Flood Feature 1 (1) ____
- ○ Hydrological Flood Feature 2 (2) ____

Do you have any other comments you would like to add?

____

____

____

____

____

Thank you for completing the survey. Don't forget to click the yellow arrow to finalise your responses and submit the survey.

Supplementary File S2: Community Development and Sustainability in Flood Disaster Management: Post-workshop Series Survey

Introduction

Thank you for agreeing to complete this post-workshop survey. De-identified responses to this survey will be used to evaluate and improve future iterations of this workshop. This study has received ethical approval from the University of Southern Queensland (H21REA073). In participating in the survey, there are no anticipated risks beyond normal day-to-day living. However, sometimes thinking about the sorts of issues raised in the questionnaire can create some uncomfortable or distressing feelings. If you need to talk to someone about this immediately, please contact the South African Depression and Anxiety Group (SADAG) on (0800) 121314 of a local hotline in your country.

By answering 'yes' below, you are indicating:

You have read and understood the information document regarding this project. Have had any questions answered to your satisfaction. Understand that if you have any additional questions, you can contact the research team. You are over 18 years of age. You understand that any data collected may be used in future research activities. You agree to participate in the project.

Please click yes to start the survey. If you don't wish to participate please exit the survey now by closing the page.

Yes (1)

Demographics and personal flood experience

Which country do you live in?

South Africa (1)
Cameroon (2)
Senegal (3)
Uganda (4)
Senegal (5)
United Kingdom (6)
Monaco (7)
Botswana (8)
Argentina (9)
Tanzania (10)
Lesotho (11)
Germany (12)
Other (please specify) (13) ____

What is your gender?

Male (1)
Female (2)
Non-binary / third gender (3)
Prefer not to say (4)

What is your age?

____

What type of organisation do you work for? [choose most appropriate]

- ○ Government (1)
- ○ Non-government organisation (2)
- ○ Emergency response (3)
- ○ Volunteer (4)
- ○ Other [please specify] (5) ____

What is your position within your organisation?

- ○ Operator/Technician (1)
- ○ Middle Manager (2)
- ○ Strategic Manager (3)
- ○ Other [please specify] (4) ____

What role(s) does your organisation play in flood management/response in your country? [select all that are relevant]

- ☐ Community Preparedness (1)
- ☐ Search & Rescue (2)
- ☐ Flood Management (3)
- ☐ Other [please specify] (4) ____

Have you responded to flooding in your country before?

- ○ Yes (1)
- ○ No (2)

Individual participants' response to training

How many of the four webinar sessions did you attend? (Please list number)

____

Overall, how would you rate the training you received?

- ○ Excellent (1)
- ○ Above average (2)
- ○ Average (3)
- ○ Below average (4)
- ○ Very poor (5)

How would you rate the training received on each topic? (from excellent to very poor or did not attend this session)

	Excellent (1)	Very good (2)	Neutral (3)	Poor (4)	Very poor (5)	Did not attend this session (6)
Flood disaster and future (1)	○	○	○	○	○	○
Emergency management (2)	○	○	○	○	○	○
Four phases of flooding (3)	○	○	○	○	○	○
Incident command-TEMPOE (4)	○	○	○	○	○	○
Flood hydrology (5)	○	○	○	○	○	○
Operations safety (6)	○	○	○	○	○	○
Training standards (7)	○	○	○	○	○	○
Equipment standards (8)	○	○	○	○	○	○

How has your knowledge on the following topics changed as a result of the training? Please choose from much better to much worse. If you did not attend the session during which a topic was covered please select 'Did not attend this session'

	Much better (1)	Somewhat better (2)	Stayed the same (3)	Somewhat worse (4)	Much worse (5)	Did not attend this session (6)
Flood disaster and future (1)	○	○	○	○	○	○
Emergency management (2)	○	○	○	○	○	○
Four phases of flooding (3)	○	○	○	○	○	○
Incident command - TEMPOE (4)	○	○	○	○	○	○
Flood hydrology (5)	○	○	○	○	○	○
Operations safety (6)	○	○	○	○	○	○
Training standards (7)	○	○	○	○	○	○
Equipment standards (8)	○	○	○	○	○	○

Would you recommend this type of training to others involved in flood?

○ Definitely would (1)

○ Probably would (2)

○ Probably won't (3)

○ Definitely won't (4)

Having completed the training, how do you rate the strengths of your individual flood response/management capacity?

- ○ Excellent (1)
- ○ Good (2)
- ○ Fair (3)
- ○ Poor (4)
- ○ Very Poor (5)

What are 3 key challenges you as an individual continue to face in flood response/management?

- ○ Challenge 1 (1) ____
- ○ Challenge 2 (2) ____
- ○ Challenge 3 (3) ____

Has the training prompted you to make any changes in the way you currently manage and/or respond to flooding?

- ○ Yes (1)
- ○ No (2)

If yes, what changes have you made or do you plan to make?

____

To what extent has your participation in the training provided you with the motivation and confidence to get involved in flood interoperability?

- ○ To a great extent (2)
- ○ Somewhat (4)
- ○ Very little (5)
- ○ Not at all (6)

After having participated in the training, how important is the value you place on collaboration with other organisations or sectors for flood mitigation or response?

○ Very important (1)

○ Important (2)

○ Moderately important (3)

○ Slightly important (4)

○ Not important (5)

If you had government financial support, what would spend the funding on? Rank in order of preference from 1 (most likely to spend on). Make your choice by dragging and dropping the options in order.

______ Training technicians (1)
______ Equipment (2)
______ Training instructors (3)
______ Planning (4)
______ Policy (5)
______ Flood defences (i.e. sea walls) (6)
______ Other please specify (7)

What topics do you still need further training on?

____

Individual respondent's knowledge

Are you familiar with the Sendai Framework?

○ Yes (1)

○ No (2)

○ Don't know (3)

In your own words, what do you think the Sendai Framework is

____

____

____

____

____

How is your work aligned to the Sendai Framework?

____

____

____

____

____

Are you aware of the coastal sea level rises that are predicted in this century?

- ○ Yes (1)
- ○ No (2)
- ○ Don't know (3)

If yes, do you know by what height sea levels are expected to rise?

____

Are you aware of any plans for mitigation or response to coastal inundation?

- ○ Yes (4)
- ○ No (5)

Name the 5 flood rescue modules

- ○ Module 1 (1) ____
- ○ Module 2 (2) ____
- ○ Module 3 (3) ____
- ○ Module 4 (4) ____
- ○ Module 5 (5) ____

Name the 4 phases of floods

- ○ Phase 1 (1) ____
- ○ Phase 2 (2) ____
- ○ Phase 3 (3) ____
- ○ Phase 4 (4) ____

Name 3 emergency flood principles

- ○ Principle 1 (1) ____
- ○ Principle 2 (2) ____
- ○ Principle 3 (3) ____

Name 2 hydrological flood features

- ○ Hydrological Flood Feature 1 (1) ____
- ○ Hydrological Flood Feature 2 (2) ____

Organisational flood mitigation and response capacity
Now thinking about your organisation.
Having now completed the training, how do you rate the strengths of your organisation's flood response/management capacity?

- ○ Excellent (1)
- ○ Above Average (2)
- ○ Average (3)
- ○ Below Average (4)
- ○ Very Poor (5)

Having completed the training, how do you view your organisational risk against flood disaster?

- ○ Very high (4)
- ○ Above average (8)
- ○ Average (9)
- ○ Below average (11)
- ○ Very low (12)

What are three key challenges your organisation still faces in flood response/management?

- ○ Challenge 1 (1) ____
- ○ Challenge 2 (2) ____
- ○ Challenge 3 (3) ____

Country-level Flood Mitigation and Response Capacity
Now thinking about your country-level flood mitigation and response capacity.

Having now completed the training, how do you rate the strengths of your country's flood response/management capacity?

- ○ Excellent (1)
- ○ Above Average (2)
- ○ Average (3)
- ○ Below Average (4)
- ○ Very Poor (5)

Having completed the training, what are three key challenges your country still faces in flood response/management?

- ○ Challenge 1 (1) ____
- ○ Challenge 2 (2) ____
- ○ Challenge 3 (3) ____

Any additional feedback?
Do you have any other comments you would like to add about the training?

____

____

____

Do you have any other comments you would like to add about flood response/management?

____

____

____

Thank you for completing the survey. Don't forget to click the yellow arrow to finalise your responses and submit the survey.
